# Vaping-Induced Lung Injury: An Uncharted Territory

**DOI:** 10.7759/cureus.8970

**Published:** 2020-07-02

**Authors:** Anchit Bharat, Nikita Jain, Belaal Sheikh, Hafiz Muhammad Jeelani, Maryna Shayuk

**Affiliations:** 1 Internal Medicine, Indiana University Health Ball Memorial Hospital, Muncie, USA; 2 Internal Medicine, Chicago Medical School, Rosalind Franklin University of Medicine and Science, McHenry, USA; 3 Internal Medicine, Northwestern Medicine McHenry Hospital, McHenry, USA; 4 Internal Medicine, Rosalind Franklin University of Medicine and Science, McHenry, USA

**Keywords:** vaping, vaping-induced lung injury

## Abstract

Vaping-associated lung injury (VALI) presents with symptoms ranging from lower respiratory tract involvement (shortness of breath, fever, and cough) to gastrointestinal involvement (vomiting and diarrhea). Based on the longitudinal analysis, VALI is associated with increased risk for respiratory disease, making it paramount for medical professionals to understand this disease process and be familiar with its varied presentations. Our case study is a presentation of two relatively young patients with VALI, with a varied clinical course and distinct levels of severity. VALI still remains uncharted territory. Case reports, such as ours, have the potential to invoke randomized controlled clinical trials to better understand the disease etiology, pathology, and management.

## Introduction

Throughout the history of smoking, nicotine delivery has always been a centerpiece of policy and industry. Efforts to modify nicotine delivery are as old as nicotine use itself. Based on a Centers for Disease Control and Prevention report in 2014, more than 16 million Americans are reported to be living with diseases caused by smoking. It is responsible for taking approximately 480,000 American lives per year. Not only in the United States (US) but worldwide as well, tobacco use causes havoc - more than seven million people lose their lives to smoking every year, which is projected to go up to eight million by 2030 (World Health Organization (WHO) report, 2017). Multiple attempts have been made to invent novel modes of delivery that can protect the consumer from the deleterious effects of smoking while retaining the ‘highs’. Electronic cigarettes (e-cigarettes) were heralded as the lesser of the two evils. An e-cigarette is a device that can aerosolize nicotine and cannabis containing liquid (also known as e-liquid) by partially heating it. The process of inhaling these aerosols is called ‘vaping’. Continuous vaping is associated with a pulmonary disease that is characterized by bilateral infiltrates on chest imaging and diffuse alveolar damage on histology [1-3]. This disease claimed widespread media coverage in 2019 when a lot of American adolescents were struck by it, reaching epidemic standards [4-6]. VALI presents with a wide-ranging symptom profile, making it difficult to diagnose [3, 7]. VALI is a major risk factor for morbidity and mortality, making it important for medical professionals to understand this disease process so they can provide high-value care to their patients [8].

## Case presentation

Patient 1

**Figure 1:**
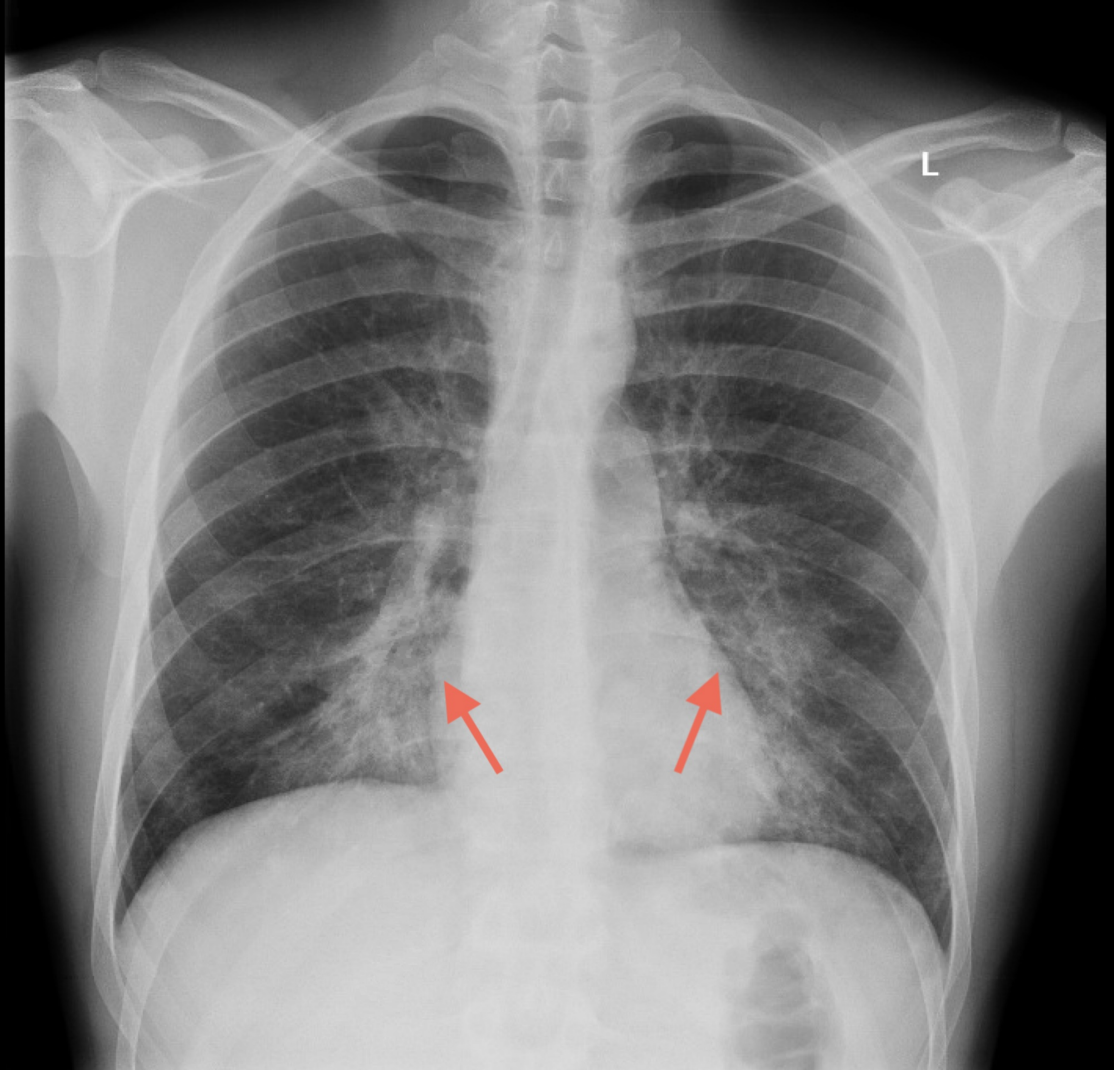
Chest x-ray (CXR) on the day of admission. Bilateral medial lung base infiltrates of uncertain chronicity were noted (red arrows).

**Figure 2:**
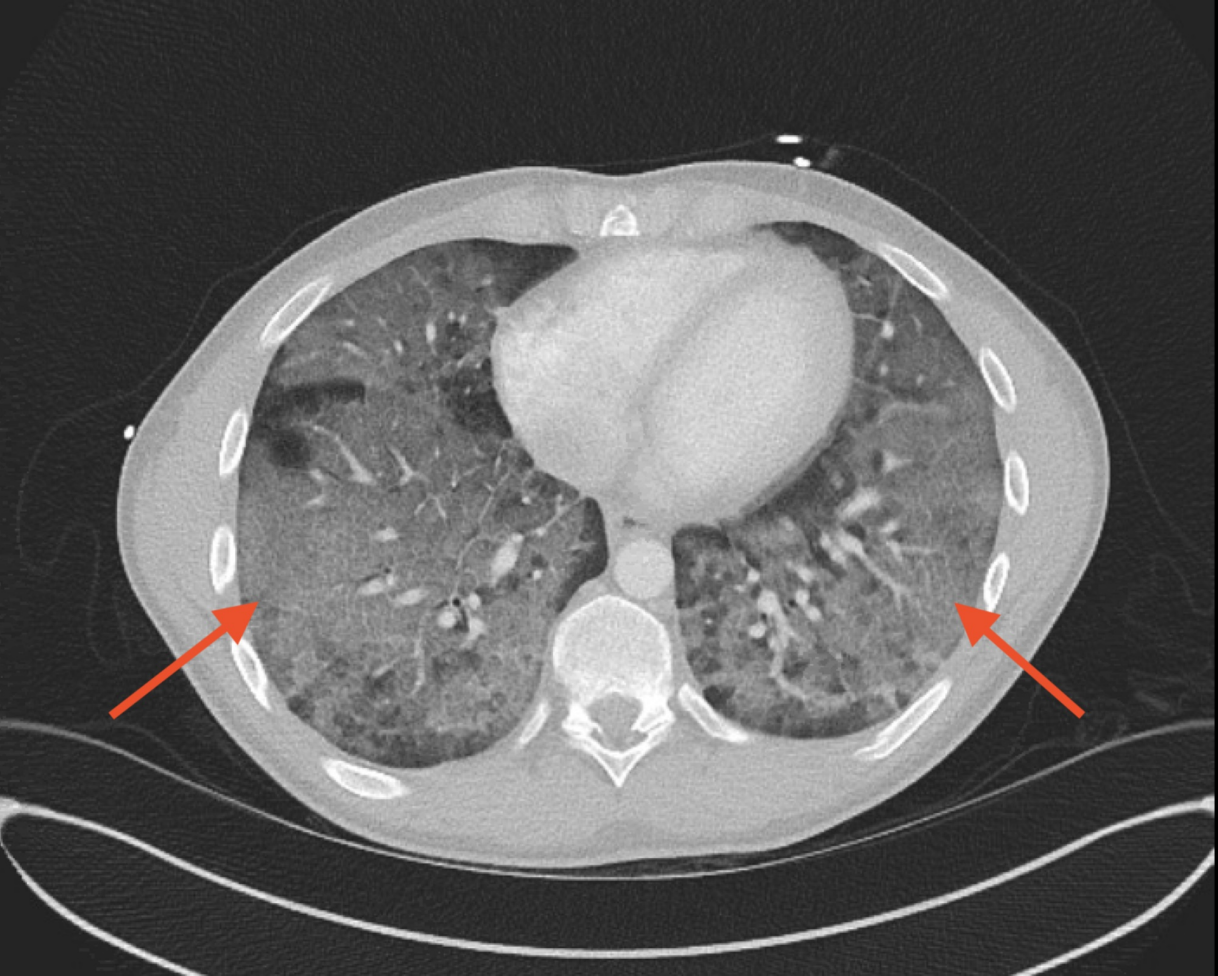
Computed tomography (CT) of the chest on Day 2 of admission with rapidly worsening ground-glass opacities throughout the lungs (red arrows).

**Figure 3:**
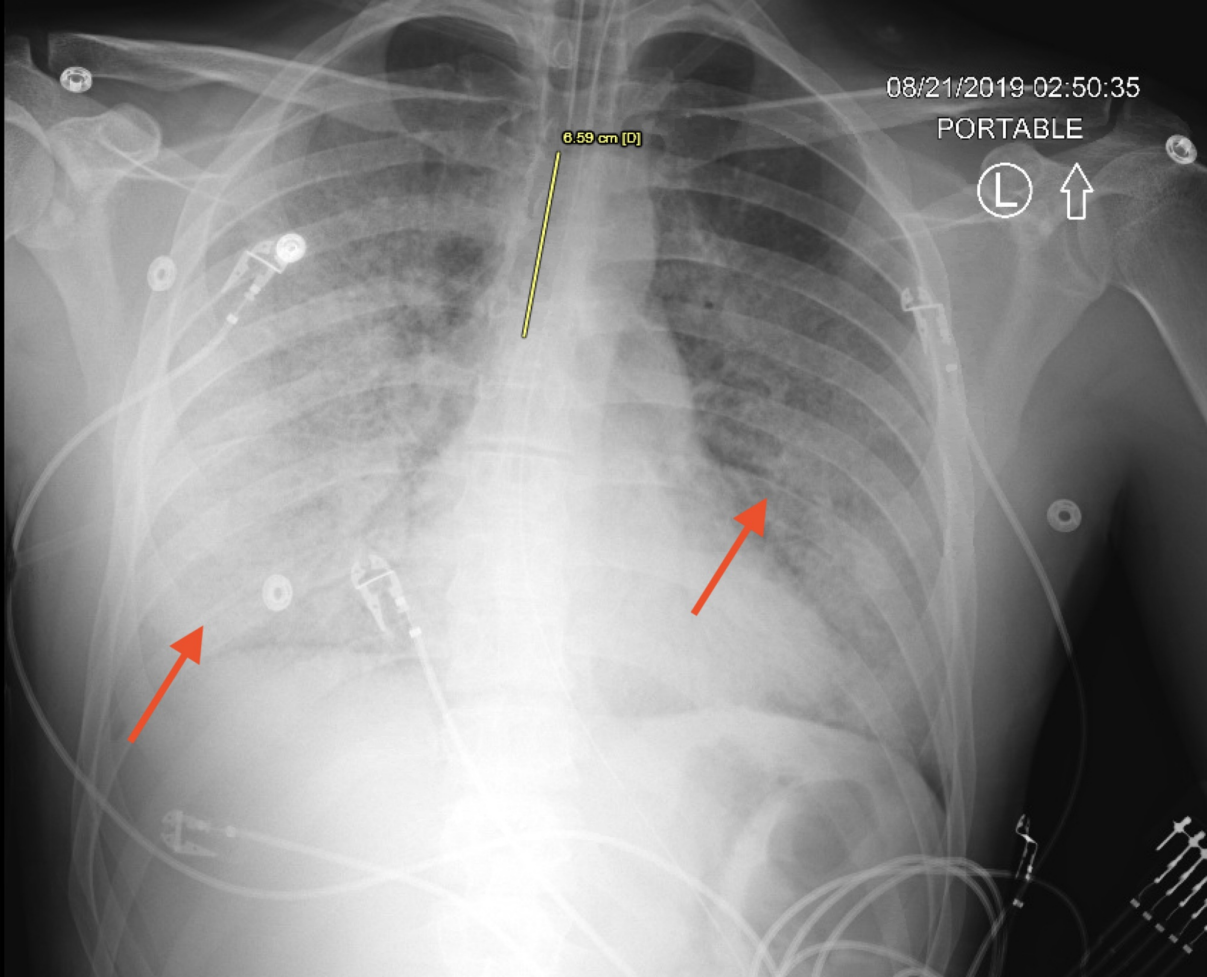
Chest x-ray (CXR) on Day 2 of admission post-intubation (yellow line showing the level of the endotracheal tube). Rapidly worsening ground-glass opacities are seen throughout the lungs with relative subpleural sparing (red arrows).

A 24-year-old male ex-smoker (quit date: one year prior) presented with a severe dry cough, sore throat, high-grade fever (38.9˚C), shortness of breath, and pleuritic chest pain for one week. Associated symptoms included chills, headaches, fatigue, myalgia, post-tussive emesis, and an episode of diarrhea. The patient tested negative on the rapid strep test (RST) at an urgent care facility and failed a five-day course of amoxicillin-clavulanate prescribed for community-acquired pneumonia before presenting to our emergency room (ER). No exposure to sick contacts, contaminated food, or a history of prolonged immobilization was reported. Physical exam revealed tachycardia (heart rate (HR) 104 beats per minute (bpm)); otherwise, his vital signs were normal and there was no cardiopulmonary distress. Labs were remarkable for a white blood cell (WBC) count of 28,800/UL with 8% bands, lactic acid - 3.9 mmol/L, total bilirubin - 1.3 mg/dL, serum aspartate aminotransferase (AST) - 44 U/L, and a normal alanine aminotransferase (ALT). Chest x-ray (CXR) revealed bilateral medial lung base infiltrates (Figure 1). Blood cultures and atypical bacterial antigens for Legionella pneumoniae, Streptococcus pneumoniae, and Mycoplasma pneumoniae were sent, and the patient was admitted to the inpatient unit for sepsis due to community-acquired pneumonia (CAP). Intravenous (IV) fluids and levofloxacin were initiated empirically. Of note, a urine drug screen was positive for cannabinoids and opiates; however, the patient denied any illicit drug use. Over the next 24 hours, the patient continued to have shortness of breath, coughing spells with emesis, and mild hemoptysis. His core temperature rose and the patient became hypoxic, requiring 10 liters of oxygen via nasal cannula. Chest computed tomography (CT) angiogram ruled out pulmonary embolism and revealed ground-glass opacities throughout the lungs with subpleural sparing, worsened compared to his previous CXR (Figures 2, 3).

**Figure 4:**
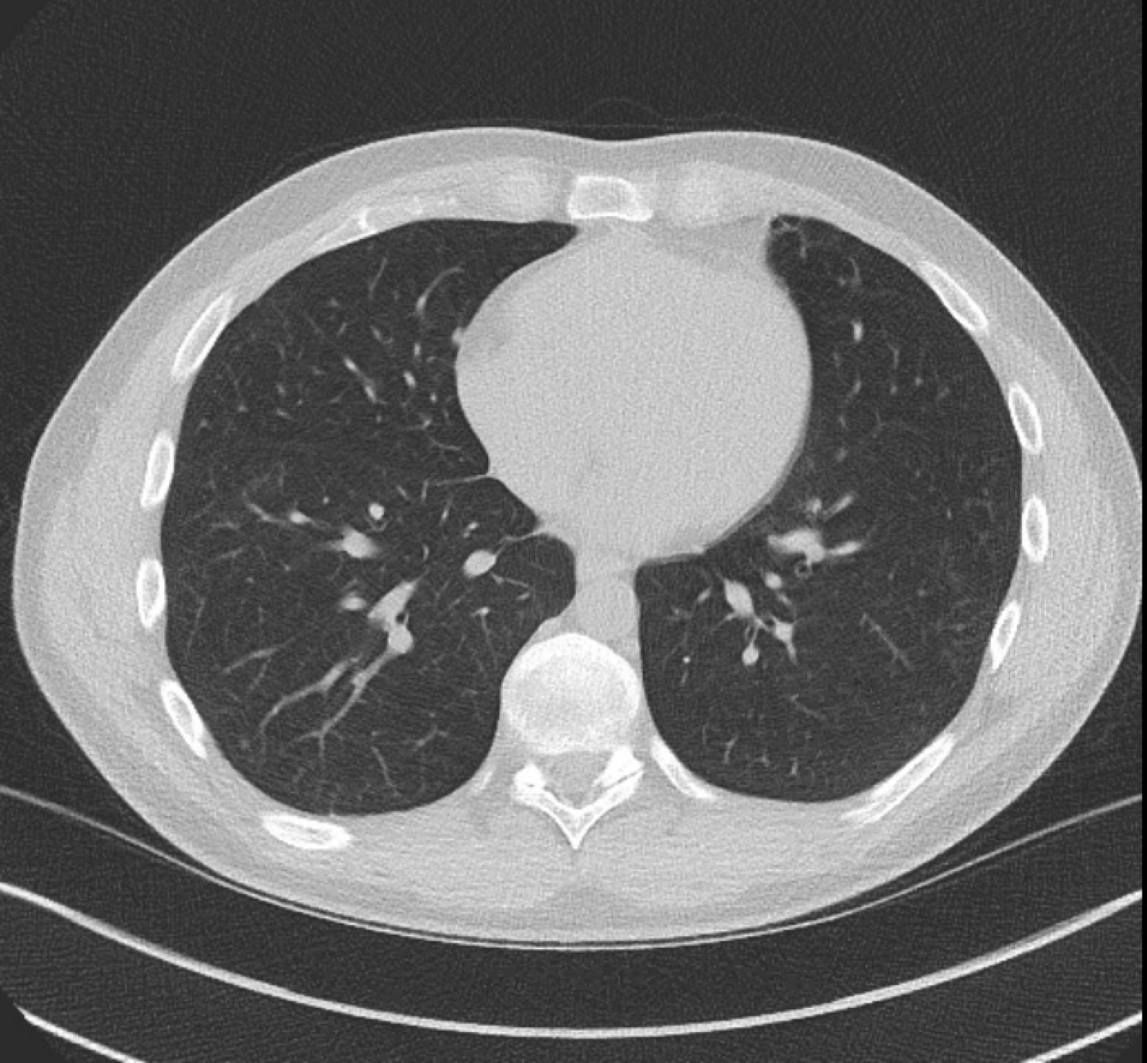
Follow-up computed tomography (CT) of the chest six weeks following discharge showing peribronchovascular ground-glass opacities/nodules in the right lower lobe that were likely due to resolving infection.

Due to a rapid decline in his clinical condition and anticipated progression to acute respiratory distress syndrome (ARDS), the patient was transferred to the intensive care unit (ICU). Arterial blood gas revealed a pH - 7.30, partial pressure of carbon dioxide (pCO_2_) - 58, partial pressure of oxygen (pO_2_) - 146, and bicarbonate (HCO_3_) - 28, suggestive of acute respiratory acidosis. In consultation with infectious disease, antimicrobials were escalated to vancomycin, double pseudomonal coverage with levofloxacin and piperacillin-tazobactam, oseltamivir for influenza, and trimethoprim-sulfamethoxazole for possible opportunistic infections, such as Pneumocystis pneumonia, while awaiting further infectious workup. On extensive follow-up history taking, the patient admitted to smoking marijuana through e-cigarettes, which he had quit two weeks prior to admission. Methylprednisolone was added for suspected VALI. The patient was placed on bilevel positive airway pressure (BiPAP) support and eventually required intubation and mechanical ventilation. Over the next few days, the patient’s respiratory status worsened, requiring high positive end-expiratory pressure (PEEP) and fraction of inspired oxygen (FiO_2_) support (PO_2_/FiO_2_ - 60). The patient was placed in a RotoProne® bed (Arjo, Inc., Addison, IL) to help with oxygenation. Serologies for blastomycosis, histoplasmosis, human immunodeficiency virus (HIV) 1 and 2 antigens, Western blot, CD4 and CD8 counts, quantiferon gold tuberculosis, blood cultures, and influenza A and influenza B viral ribonucleic acid (RNA) were negative, as were bronchoalveolar lavage cultures for gram stain, fungi, viruses, acid-fast bacilli, and Legionella. The differential for bronchoalveolar lavage revealed a total cell count of 200 with 82% neutrophils, 9% lymphocytes, 7% monocytes, and 2% macrophages. Additionally, normal transthoracic echocardiogram ruled out congestive heart failure, pulmonary hypertension, and valvular disorders. The diagnosis of severe ARDS secondary to VALI was made. Antibiotics were discontinued and the patient was managed with high doses of steroids with subsequent improvement in his overall clinical condition. The e-cigarettes were seized and Infection Control was notified per the hospital policies. The patient was extubated, weaned off supplemental oxygen to room air, and was discharged home with a steroid taper over 3.5 weeks. Follow-up imaging six weeks after discharge showed significant improvement as shown in Figure 4. 

Patient 2

**Figure 5:**
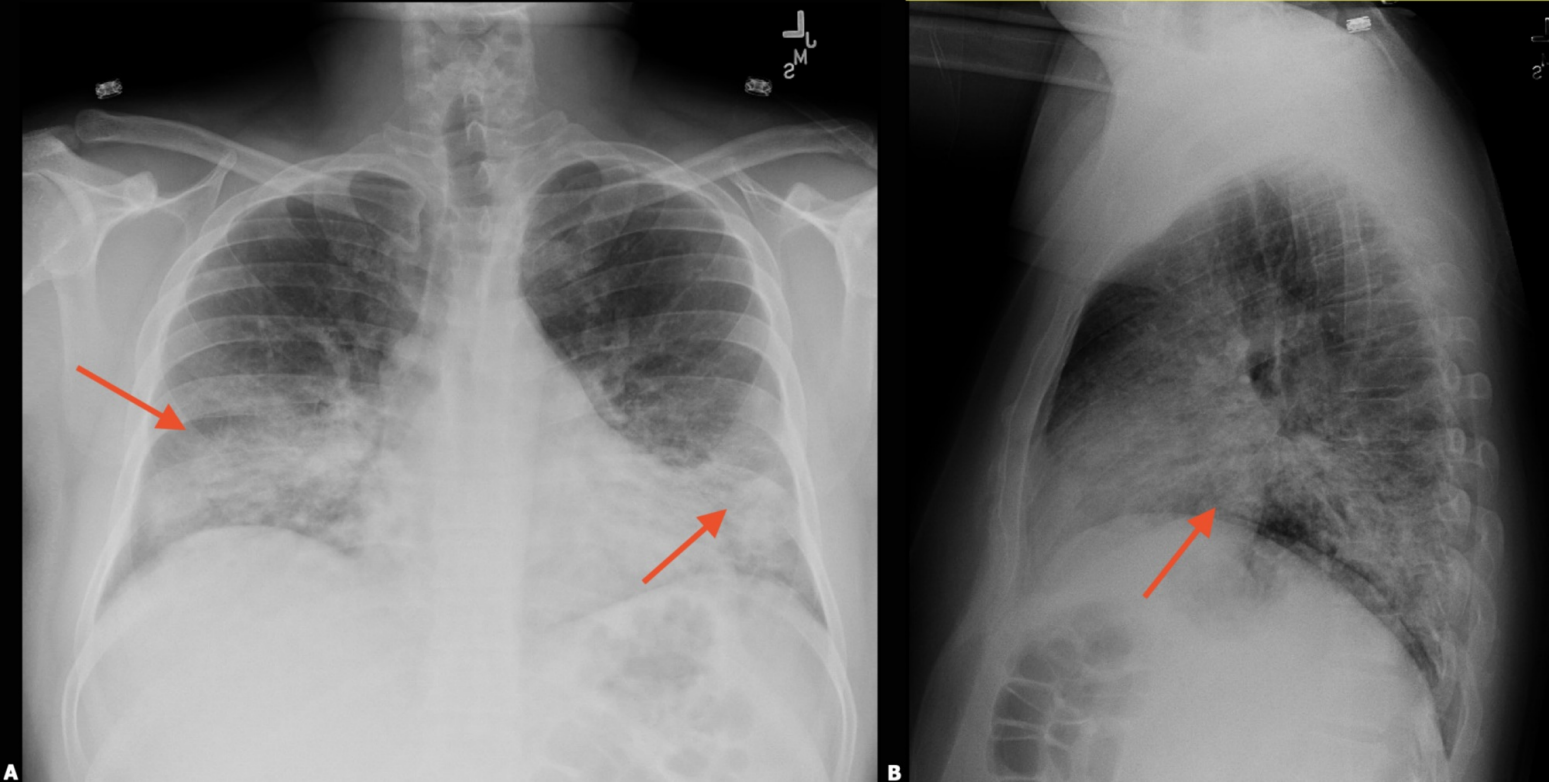
CXR – day of admission showing bilateral lower lobe interstitial infiltrates (red arrows). (A): Postero-anterior view; (B): Lateral view.

A 38-year-old male with no significant past medical history presented to the ER with complaints of exertional dyspnea associated with flu-like symptoms, including low-grade fevers, chills, occasional episodes of diarrhea, chest pain, and cough productive of yellow to gray sputum for two weeks. The patient initially went to an urgent care facility five to six days prior to admission where he was diagnosed with pneumonia (CXR not available). The patient completed a course of azithromycin and amoxicillin-clavulanate without improvement in symptoms and thus presented to the ER. He reported exposure to sick contacts (children with an upper respiratory infection) and a history of vaping until two to three weeks prior to admission, which he stopped due to the symptoms. History was unremarkable for any exposure to birds, occupational exposures, immunocompromised status, outdoor activities, or recent travel. On presentation, vital signs were remarkable for temperature 38.2°C, heart rate of 115, respiratory rate 33, and oxygen saturation of 76% requiring 3 liters of oxygen via nasal cannula. Lung examination revealed rhonchi on bilateral bases with associated egophony. Laboratory studies revealed a WBC count of 18.3 with left shift, thrombocytosis, and a lactic acid level of 2.2 mg/dL. CXR demonstrated bilateral lower lobe consolidation (Figure 5). 

**Figure 6:**
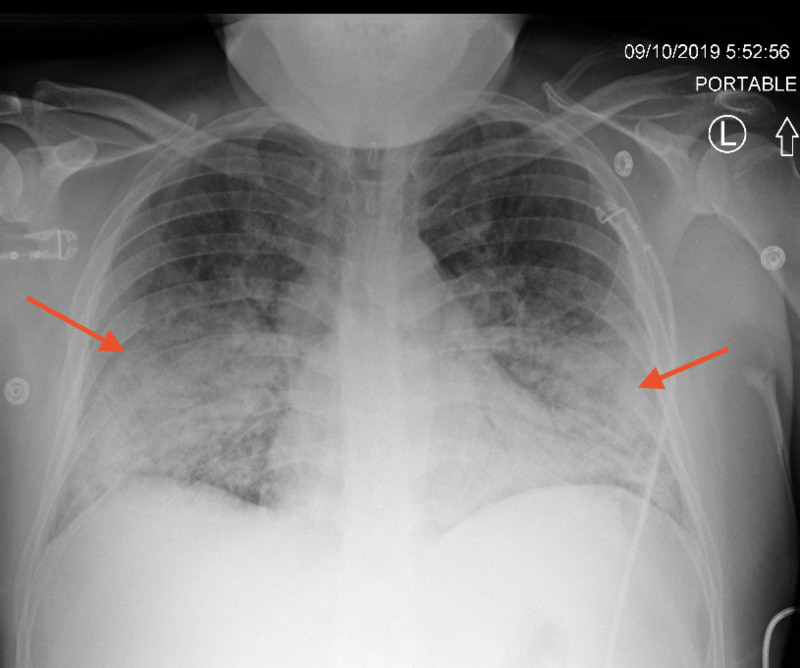
Chest x-ray on Day 3 of admission showed rapidly worsening bilateral lower lobe infiltrates with subpleural sparing (red arrows).

**Figure 7:**
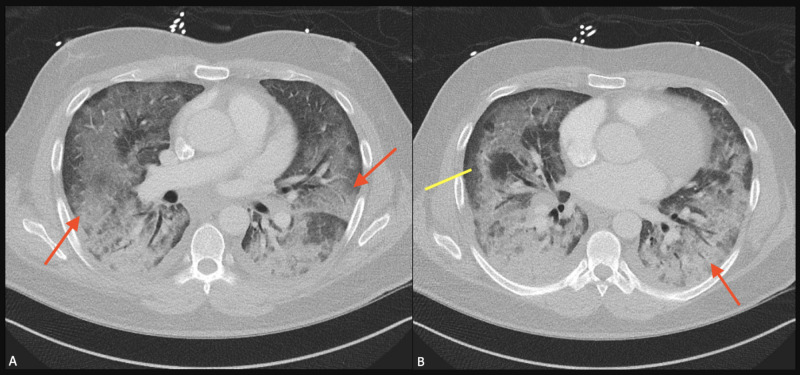
Computed tomography of the chest on Day 3 of admission showed (A) rapidly worsening bilateral lower lobe infiltrates (red arrows) with (B) subpleural sparing (yellow line) and worsening lower lobe infiltrate (red arrow).

The patient was admitted for severe sepsis secondary to bilateral pneumonia and was initiated on levofloxacin, which was later changed to vancomycin and ceftriaxone. Infectious disease was consulted due to the failure to respond to antibiotics and the possibility of fungal etiology. Over the course of the next few days, the patient did not show significant improvement. Imaging was repeated and included a CT scan of the chest, along with a CXR, for a more detailed evaluation of the lung parenchyma. This showed a rapidly worsening airspace opacities (Figures 6, 7). Antibiotics were adjusted to include doxycycline, and ceftriaxone (dose was increased to 2 g every 24 hours). Work-up, including streptococcal pneumonia antigen, Legionella urinary antigen, Mycoplasma pneumonia, blood cultures, sputum cultures, and methicillin-resistant staphylococcus aureus (MRSA) nasal swab, was negative. A procalcitonin level was 0.18 ng/mL, as well as a C-reactive protein at 27.8 mg/dL.

**Figure 8:**
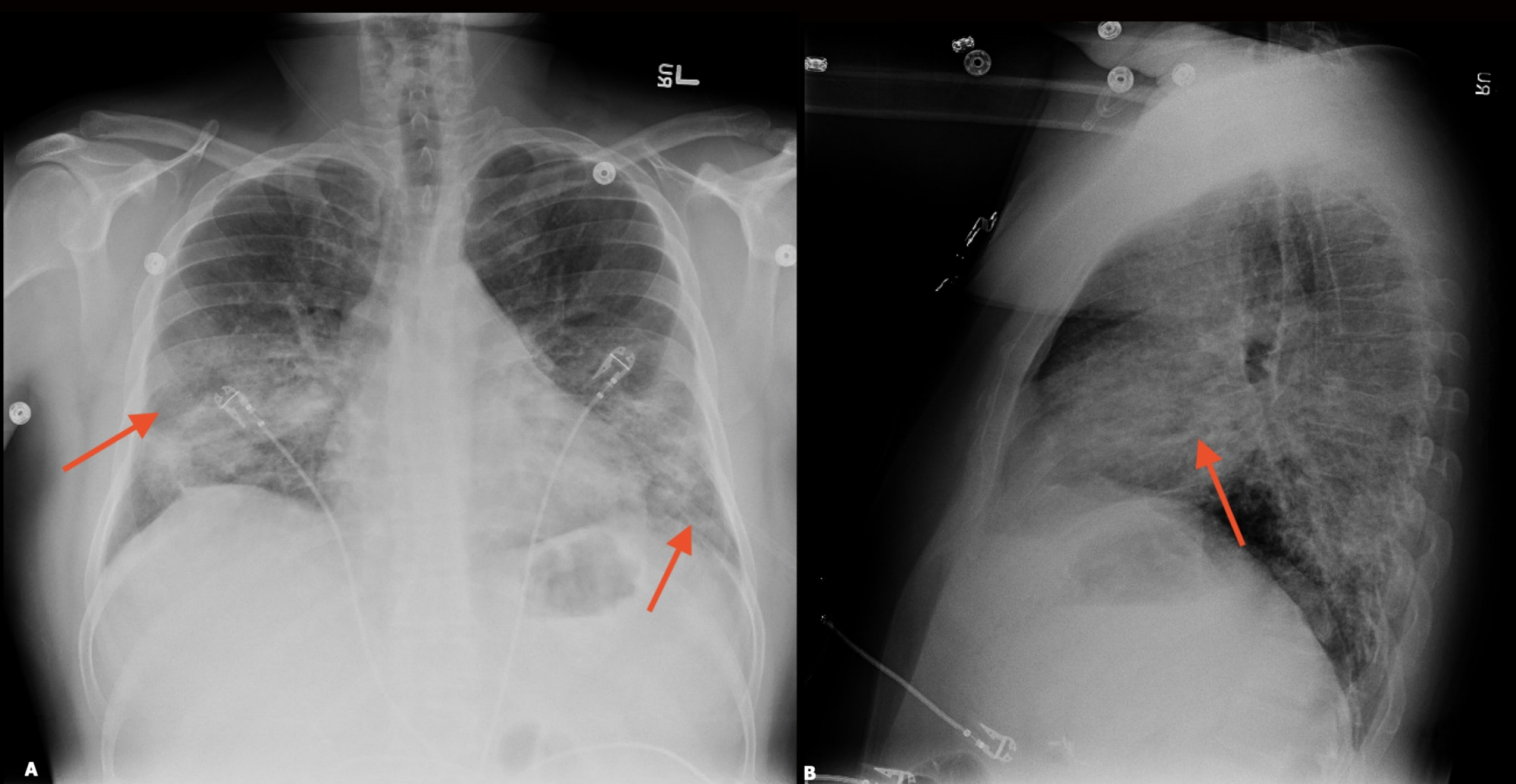
Chest x-ray after the initiation of steroids Interval improvement was seen in bilateral airspace opacities (red arrows). A) Postero-anterior view; B) lateral view

**Figure 9:**
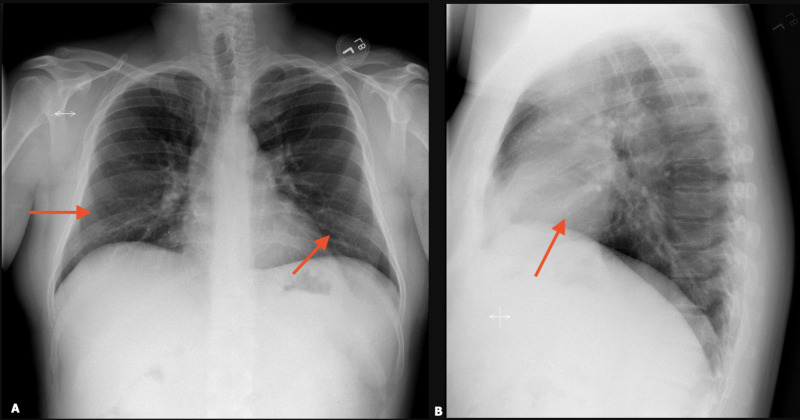
Follow-up chest x-ray two weeks following discharge showed almost complete resolution of bilateral airspace opacities with near normal lung parenchyma (red arrows) A) Postero-anterior view; B) lateral view

Due to a lack of improvement, persistent fevers, increasing oxygen requirements up to 8 liters via nasal cannula, and worsening leukocytosis, pulmonary service was consulted. The patient underwent a bronchoscopy, which revealed pitting of the bronchial mucosa due to bronchitis. Bronchoalveolar lavage was sent for culture, cytology, differential, bacterial, and fungal studies which came back negative. The patient was started on methylprednisolone, 0.5 mg/kg twice daily, with remarkable improvement (Figure 8). Antibiotics were discontinued, the patient was weaned off oxygen, and discharged home with a steroid taper over four weeks. A follow-up CXR two weeks following discharge revealed marked improvement in airspace opacities to almost complete resolution (Figure 9).

## Discussion

Herein, we contrast two different patients who were treated at our facility. Except for a lack of underlying comorbidities, symptomatology, the use of e-cigarette (marijuana products), and radiological findings, they had nothing in common. The severity of disease was greater in Patient 1 than Patient 2, despite being younger. There was no history of high-risk sexual behavior, intravenous drug use, spelunking, or exposure to birds. The repeated denial of using illicit drugs by Patient 1 contributed to a delay in diagnosis. His disease severity remains a medical mystery to this date. Both of our patients received empiric antibiotics initially which were later discontinued due to lack of response. Both received intravenous steroids with marked improvement in their symptoms. Both were able to quit using e-cigarettes. Partial resolution of symptoms was noted within three to four weeks. With our cases, we present two different VALI severities to give clinicians insight on what to expect throughout the hospital course. The importance of an extensive clinical history is emphasized. If not for our extensive history, Patient 1's diagnosis might have been substantially delayed, leading to a lag in steroid initiation, thereby causing adverse events. The importance of steroids should be noted. Appropriate follow-up with a key emphasis on imaging is stressed within four to six weeks of discharge. Patients should be counseled to stay off e-cigarette usage for sustained positive outcomes. 

## Conclusions

VALI still remains uncharted territory. Case reports such as these have the potential to invoke randomized controlled clinical trials to better understand the disease etiology, pathology, and management. Our case study highlights the common clinical and radiological features of VALI with different severities. The pathophysiology behind this remains unknown, but the role of vitamin E might be implicated. Further studies are warranted to explore the role of steroids in controlling this disease and the appropriate dosage. Thorough history taking is the key to making a diagnosis of VALI, and this disease should be part of the differential diagnosis in acute respiratory failure in light of vaping history. 
